# ROY Crystallization on Poly(ethylene) Fibers, a Model for Bed Net Crystallography

**DOI:** 10.1021/acs.chemmater.3c03188

**Published:** 2024-02-27

**Authors:** Bryan Erriah, Alexander G. Shtukenberg, Reese Aronin, Derik McCarthy, Petr Brázda, Michael D. Ward, Bart Kahr

**Affiliations:** † Department of Chemistry and Molecular Design Institute, New York University, New York, 29 Washington Place, New York City, New York 10003, United States; ‡ Department of Structure Analysis, Institute of Physics, Czech Academy of Sciences, Na Slovance 2/1999, Prague 8 18221, Czech Republic

## Abstract

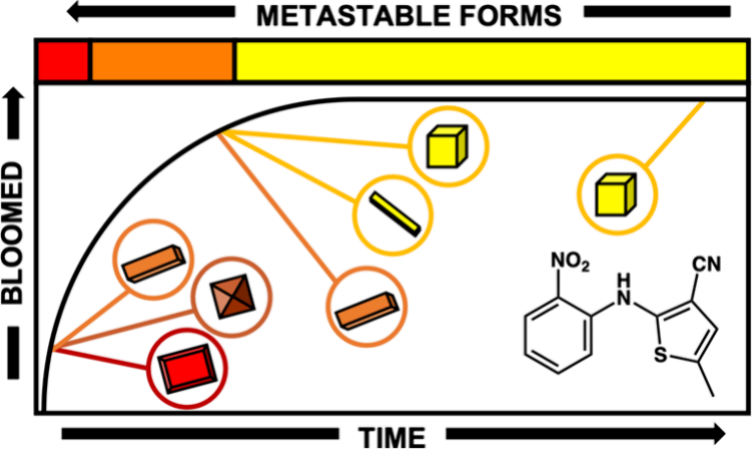

Many long-lasting insecticidal bed nets for protection against disease vectors consist of poly(ethylene) fibers in which insecticide is incorporated during manufacture. Insecticide molecules diffuse from within the supersaturated polymers to surfaces where they become bioavailable to insects and often crystallize, a process known as blooming. Recent studies revealed that contact insecticides can be highly polymorphic. Moreover, insecticidal activity is polymorph-dependent, with forms having a higher crystal free energy yielding faster insect knockdown and mortality. Consequently, the crystallographic characterization of insecticide crystals that form on fibers is critical to understanding net function and improving net performance. Structural characterization of insecticide crystals on bed net fiber surfaces, let alone their polymorphs, has been elusive owing to the minute size of the crystals, however. Using the highly polymorphous compound ROY (5-methyl-2-[(2-nitrophenyl)-amino]thiophene-3-carbonitrile) as a proxy for insecticide crystallization, we investigated blooming and crystal formation on the surface of extruded poly(ethylene) fibers containing ROY. The blooming rates, tracked from the time of extrusion, were determined by UV–vis spectroscopy after successive washes. Six crystalline polymorphs (of the 13 known) were observed on poly(ethylene) fiber surfaces, and they were identified and characterized by Raman microscopy, scanning electron microscopy, and 3D electron diffraction. These observations reveal that the crystallization and phase behavior of polymorphs forming on poly(ethylene) fibers is complex and dynamic. The characterization of blooming and microcrystals underscores the importance of bed net crystallography for the optimization of bed net performance.

## Introduction

Blooming is a migratory phenomenon wherein a component of a solid mixture after phase separation moves to the external surface through the process of diffusion.^1^ In many cases, the bloomed component then crystallizes on the surface.^2,3^ The blooming process is commonly considered to be an unintended consequence of additive incorporation and is often a major concern in industries that produce food packaging,^4−6^ biomedical devices,^1,7^ rubber,^8,9^ and flame-retardant materials.^10^ Blooming may also be engineered into materials to impart desirable qualities, however, as is the case for long-lasting insecticide nets (LLINs).^11,12^

Bed nets have been used for centuries to prevent the spread of vector-borne diseases. While the first iterations were made from materials like cotton, contemporary bed nets are manufactured from synthetic polymers and some bloom insecticides. The toxicants have been shown to increase the effectiveness of nets that are otherwise merely barriers.^13−17^ The inclusion of insecticidal compounds in nets has revolutionized the fight against diseases such as malaria; in fact, the decline in malaria mortality in the 21st century is attributed largely to long-lasting insecticidal bed nets (LLINs),^18−20^ nearly three billion of which have been distributed worldwide. The benefits of LLINs, however, are dwindling “at an alarming rate”^21^ due to the development of insecticide resistance^22−24^ in mosquito populations, mosquito behavioral changes,^25^ and syndemic health crises.^26−28^ Nevertheless, chemical interventions, including LLINs,^15−17^ are expected to be mainstays of malaria prophylaxis for at least the next decade,^29^ despite progress in vaccines^30^ and gene drive technologies.^31,32^ In the case of poly(ethylene) (PE) LLINs, “long-lasting” refers to the aging of supersaturated net fibers, wherein they continually evolve active ingredients from the fiber volume to the surface where they are contacted by mosquitoes.^33^ PE nets are manufactured via the incorporation process. This process involves mixing the polymer, active ingredients, and additives, extruding them into fibers, and finally, weaving them into a net. High- and low-density PE (HDPE and LDPE, respectively) are mixed to exploit the characteristics of each form. HDPE is typically around 90% crystalline, and LDPE is less crystalline, containing more amorphous regions. Molecules of active ingredients can easily dissolve in a molten PE; however, as temperature decreases below the PE melting point, their miscibility drops and PE becomes supersaturated with the additive.^34^ This supersaturation drives a phase separation that results in the migration of the additive to the fiber surface where it becomes bioavailable. Subsequent surface diffusion of the emerged insecticide results in the growth of crystals of the active ingredients on the bed net fibers.

Despite being the largest item in the malaria control budget and extensive research on net development and testing,^35,36^ LLINs are reportedly failing to perform as expected for the full three years in the field, a standard performance requirement of the World Health Organization (WHO).^37^ This issue was addressed at a Convening on Insecticide Treated Net (ITN) Quality and Performance in 2021, which raised questions about why the nets are not meeting expectations. Following this, WHO admitted that it does not know the form of the active ingredients in the LLINs and suggested that the focus of chemistry assessments should be shifted from total [active ingredient] content to surface concentration and bioavailability.^38^ In a subsequent Convening, experts emphasized the need for physical and chemical characterization to identify the surface chemistry characteristics that would be informative and the methods that should be developed.^39^ Scanning electron microscopy has revealed minute crystals in and on PE,^34,40,41^ but the crystalline forms (a.k.a. polymorphs) have not been identified. We are unaware of any crystallographic analysis of molecular crystal polymorphs that have bloomed on the surface of any PE bed net fiber. Since the activity of polymorphs of a particular insecticide and their corresponding amorphous forms can vary markedly,^42−46^ it is essential to know the history of crystals on the surface of PE fibers so that crystal growth can be steered toward the most effective form.

**Figure 1:**
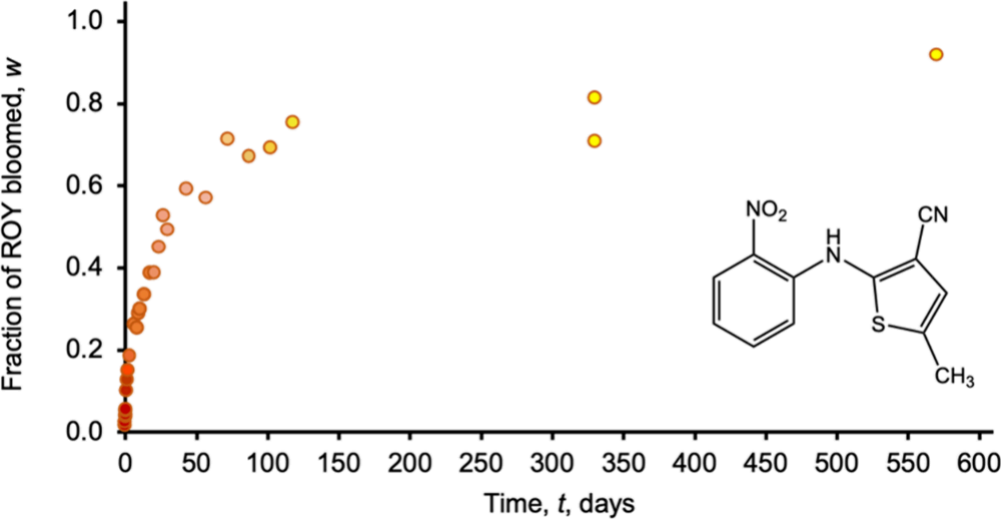
Fraction of ROY, *w*, bloomed on a fiber surface determined spectrophotometrically as a function of time, *t*. The color of the data points illustrates the color change observed in the fiber over time. The inset shows the molecular structure of ROY.

In this study, we demonstrate the fabrication of PE fibers with additives and employ Raman microscopy, scanning electron microscopy, and 3D electron diffraction (3D ED) to analyze the kinetics of blooming and the structure of the crystals that form on the surface of the extruded PE fibers. We use a model solute, ROY 5-methyl-2-[(2-nitrophenyl)-amino]thiophene-3-carbonitrile (Figure 1), as a proxy for contact insecticides. ROY (named for the Red, Orange, and Yellow colors of its polymorphs) was chosen for our initial investigation with bed nets because of its rich polymorphism.^47−49^ Moreover, the associated colors are good reporters of polymorphs, making ROY a convenient platform for studying crystal growth and phase transformations at fiber surfaces. To date, the single crystal structures of 13 ambient ROY polymorphs have been reported, and ROY is increasingly being used as a tool to study crystallization mechanisms and structure–property relationships.^50−55^ The results described herein demonstrate the dynamic nature of additives blooming from polymers and outline a framework for bed net crystallography. The results illustrate the feasibility 3D ED for routine characterization of LLIN active ingredients at the crystallographic level^56−61^ as well as methods such as Raman scattering, wherein sensitive functional groups can be used to differentiate polymorphs *in situ*. These techniques can enable entirely new areas of inquiry, including bed net crystallography, a structural inquiry that ideally would have been advanced before the distribution of three billion LLINs.

## Experimental Section

Low-density poly(ethylene) (LDPE, melt index 25 g/10 min (190 °C/2.16 kg), ρ = 0.925 g/cm^3^) and high-density poly(ethylene) (HDPE, melt index 12 g/10 min (190 °C/2.16 kg), ρ = 0.952 g/cm^3^) were purchased from Sigma-Aldrich. Polymer batches were ground in a Spex CertiPrep 6800 Freezer/Mill operated under liquid N_2_ and subsequently mixed in a weight ratio of 1:2.^12,41^ A solution of ROY (>97% TCI) in several milliliters of acetone was added dropwise to the ground polymer to achieve predetermined ratios of ROY to PE of 1 or 0.1 wt %. ROY polymorphs have melting points in the range of 62–115 °C, reaching the melting point of the PE mixture of 110–120 °C. The ROY-colored PE mixtures were placed in a 5 mL glass syringe mounted on a New Era Pump Systems syringe pump and heated using a thermokinetic heater control unit and heating pads set to 170 °C. The melt was extruded through a hole of 1 mm in diameter at a rate of 1 mL/min and pulled to approximately 500 μm in diameter while spooled on a motorized rotor. Fiber stretching was accomplished with a HP-series force gauge.

The amount of ROY bloomed on the surface after a given time was determined by storing fibers, cut to 2 cm lengths, at 30 °C in glass vials, with 2–30 pieces per vial. At various times, the vials were filled with 3 mL of absolute ethanol and agitated with a Vortex-Genie 2 for 1 min at room temperature to ensure full dissolution of ROY from the fiber surface. The fibers then were removed from the vials, and the solution was transferred to a quartz cuvette for measurement of optical absorbance (Agilent Cary 3500 UV–vis spectrometer). The concentrations of the solutions were calculated from the Beer–Lambert law (ε= 0.018 M^–1^cm^–1^ at 397 nm) based on a series of standard solutions of ROY in ethanol.

Raman spectra were collected with a Raman microscope (DXR, Thermo Fisher Scientific) using a 785 nm excitation laser operating at 10 mW, full-range grating, and a 50 μm slit width. The data were analyzed with OMNIC software.

Samples for scanning electron microscopy (SEM) were coated with 5–10 nm gold films, and crystal morphologies were recorded with a MERLIN field-emission scanning electron microscope (Carl Zeiss) using a standard Everhart–Thornley-type detector at an acceleration voltage of 5–10 kV.

3D electron diffraction (3D ED) was performed at 150 K on a FEI Tecnai G^2^ 20 microscope (200 kV, λ = 0.0251 Å) with a LaB_6_ cathode equipped with a Cheetah ASI direct detection camera (16 bit). Data were measured by continuous rotation with a rotation semiangle of 0.15°. The crystals were scratched from the fiber surface using tweezers and transferred onto the TEM grid (Cu Quantifoil carbon R 1.2/1.3 200 mesh). Data were processed with PETS2.^62^ Optical distortions were compensated using known calibrations, and camera length was calibrated using an external Lu_3_Al_5_O_12_ garnet standard.^63^ The rate of the lattice parameter change, determined for the ON polymorph, was less than one percent per 1 e^–^/Å^2^ of the deposited dose, which does not compromise the identification of the polymorph. The influence of beam damage on the lattice parameters was obtained on merged data from six crystals with a combined completeness of about 80%.

## Results and Discussion

Fibers of pure PE are colorless and translucent, whereas newly extruded fibers containing ROY are orange/red and change to yellow over time, with λ_max_ evolving from 489 to 401 nm. Blooming begins shortly after the solidification of the extruded melt, and a crust of small ROY crystals is evident on the fiber 5 h after extrusion. The fraction of ROY bloomed (relative to the initial amount of ROY in the fiber) was determined at various times by immersing individual fibers in ethanol and measuring the optical absorbance of dissolved ROY in the ethanol solution (see the Experimental Section). In the case of fibers containing 1 wt % ROY and aged at 30 °C (Figure 1), nearly 80% of the initial ROY content bloomed at the surface within 125 days, after which the rate of blooming substantially decreased. Only an additional 10% bloomed over the following 400+ days.

Blooming was simulated by assuming ROY transport throughout an HDPE/LDPE mixture is isotropic and is regulated by Fickian diffusion. Diffusion in an infinite cylinder of radius *R* is described by eq 1, where *C* is the ROY concentration, *D* is the diffusion constant assumed to be constant, and *r* is the radial distance from the center of the cylinder.
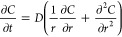
1

The initial boundary condition can be specified as *C*(*r*,0) = *C*_0_, where *C*_0_ is the concentration over all values of *r* at *t* = 0. The boundary condition at *r* = *R* corresponds to the surface evaporation model^64,65^ given by eq 2. Here, α [cm/s] is a rate constant responsible for ROY molecules crossing the fiber surface, *C*_s_ is the ROY concentration at the surface of the cylinder, and *C*_s,eq_ is its equilibrium surface concentration.
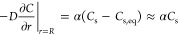
2

Crystal nucleation and growth are assumed to be very fast such that ROY molecules are assumed to diffuse through the surface of the fiber and immediately attach to the crystals or form new crystals; hence, the effective surface concentration of ROY in molecular form is very small. In this sense, crystals are a sink, and the constituent molecules in the crystals do not contribute to the concentration of ROY in molecular form at the surface. This is because surface diffusion is expected to be several orders of magnitude faster than the diffusion within the PE fiber volume^66,67^ and the formation of crystals on the fiber surface occurs within the first 5 h after the fiber fabrication. Thus, the term *C*_s,eq_ is small and can be neglected and ROY transport at the surface can be assumed to be diffusion-limited with a dimensionless parameter η = α*R*/*D* ≫ 1. The release of ROY from the interior of the fiber will cease once the volume concentration is less than the solubility of ROY in PE, *C*_eq_. The correct solution to eq 1 then requires subtraction of the solubility *C*_eq_ from the concentration *C* for all *t* values. The solubility of ROY in PE is *C*_eq_ ≪ 0.1% because even this concentration results in fast blooming (Figure 3g–i). This value is much smaller than the initial concentration *C*_0_ = 1%, obviating the necessity of this correction.

The fraction of the initial ROY concentration in the fiber that is released onto the surface and stored in the form of crystals, *w*, can be calculated explicitly with Bessel functions.^68^ The direct fitting of experimental data is not straightforward because the values of *D* and α are not known, however. Therefore, a previously reported solution was used that relied on dimensionless parameters η and .^69^ For η ≫ 1 and experimentally determined fractions *w* < 0.85 (except one point corresponding to 570 days), this solution can be written in the form of eq 3
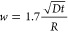
3

**Figure 2:**
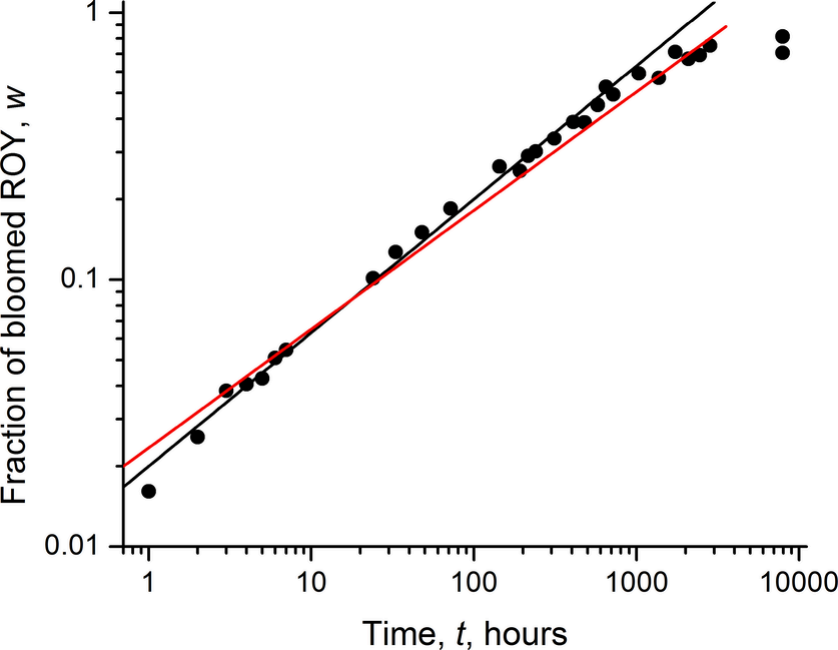
Fraction of bloomed ROY versus time (hours) replotted on a log–log scale. The black line is fit to eq 4. The red line is fit to eq 5.

Experimentally measured fractions *w* plotted versus *t* (in hours) on a log–log scale reveals a linear relationship (Figure 2), fitted by eq 4. The exponent (0.44) is very close to 0.5, which is characteristic of diffusion-limited transport, with the corresponding fit provided by eq 5
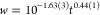
4
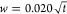
5

**Figure 3:**
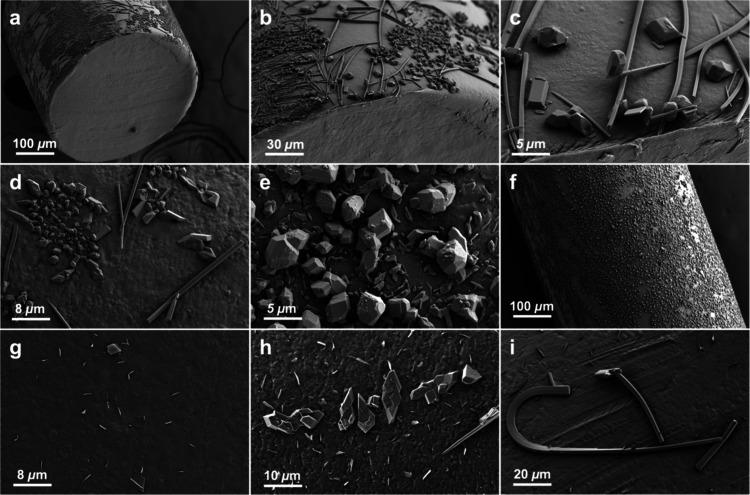
SEM images of PE fibers containing ROY. (a–c) Cross section and surface of 1% ROY fiber 7 days after production under increasing magnification demonstrating block, plate, and needle morphologies. (d) 1% ROY PE fiber 2 days after production, (e, f) 1% ROY PE fiber 330 days after production, (g, h) 0.1% ROY PE fibers 2 and 28 days after production, respectively, (i) bent, fishhook-like, needle as an example of crystal morphologies seen on 0.1% ROY fibers approximately 1 month after production.

Equations 3 and 5 afford a diffusion coefficient of *D* = 3.5 × 10^–11^ cm^2^/s for ROY in PE for the fiber diameter 2*R* = 0.6 mm. This value is approximate because η is not known, the free surface area accessible is decreasing with time (see, e.g., Figure 3d,e), and the fitting of experimental *w*(*t*) data is imperfect. Nonetheless, this value is comparable to diffusion coefficients of small molecules in polyolefins^68,69^ and supports the blooming model used here.

To date, ROY has exhibited 13 polymorphs, 12 of which are associated with solved single crystal structures.^53,70^ Scanning electron microscopy (SEM) 2 days after extrusion of PE fibers containing 1% ROY presented as crystals with at least three distinct morphologies—needles, plates, and near-isometric polyhedra—in comparable amounts and up to 5 μm in size (Figure 3d–f). The fiber surface was dominated by the polyhedra after five months (Figure 3e,f), indicative of phase transformations from the needle and plate forms. Similar morphologies were observed in fibers containing 0.1% ROY, but there were fewer crystals and they transformed more slowly (Figure 3g,h). Long needles were dominant on fibers containing 0.1% ROY after 1 month (Figure 3i).

**Figure 4:**
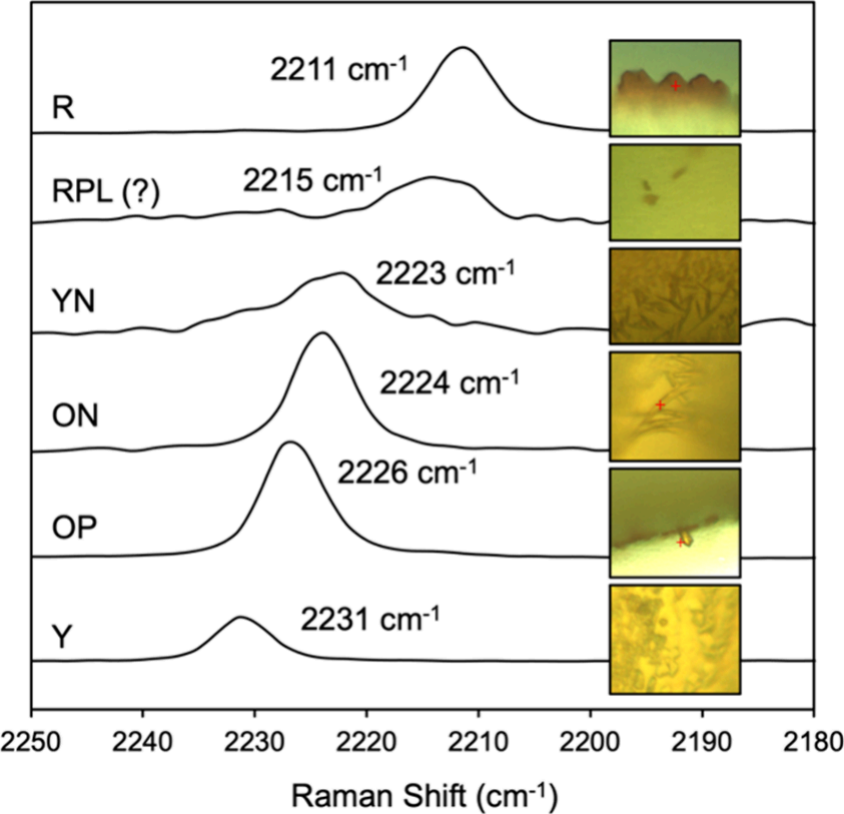
Raman spectra of ROY polymorphs observed on PE fibers. From top to bottom: R, RPL (tentatively), YN, ON, OP, and Y. Insets: optical micrographs of crystals taken under the Raman microscope.

**Figure 5:**
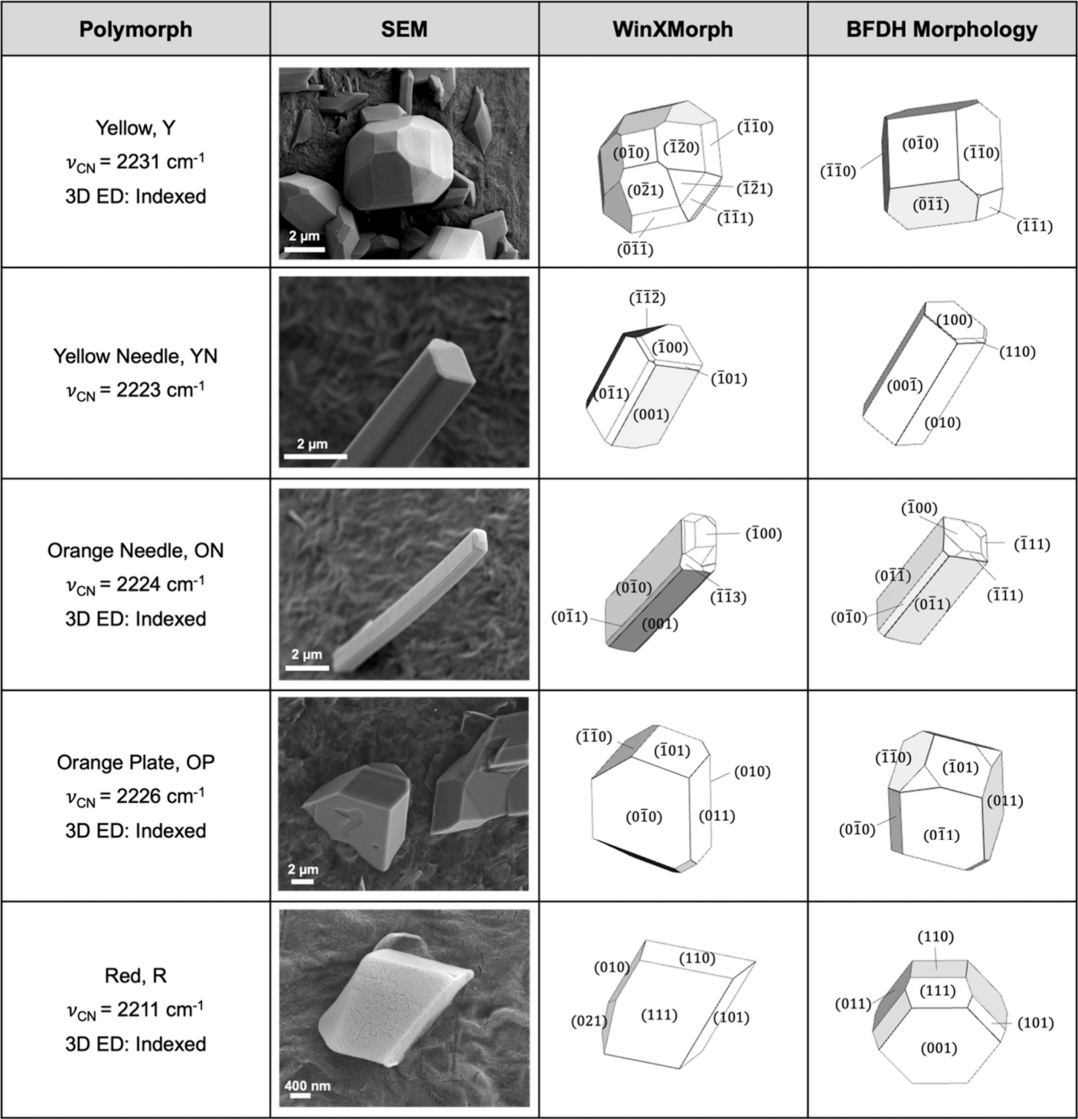
SEM (column 2), WinXMorph^70^ idealized morphology (column 3), BDFH morphology (column 4). Row 1: Y blocks. Row 2: YN needles. Row 3: ON needles. Row 4: OP plates. Row 5: R blocks/plates.

The ROY polymorphs were identified by color, morphology, and Raman-active nitrile stretching vibrations (ν_CN_), a functional group also present in many synthetic pyrethroid insecticides, by far the most common class of contact insecticides in bed nets.^53^ At least six crystalline forms were identified on the surface of the PE fibers. The dominant yellow polymorph (Y) was assigned from its ν_CN_ = 2231 cm^–1^ (Figure 4). The crystal habit matches the Bravais–Donnay–Friedel–Harker (BDFH) morphology well, and the match was refined using WinXMorph software (Figure 5).^71^ Yellow needles (YN, Figure 5) were assigned from its ν_CN_ = 2222 cm^–1^ (Figure 4). Melt crystallization of ROY performed in parallel revealed that YN readily converts to Y, a transformation that is apparent on the fibers containing 1% ROY from the eventual dominance of Y (Figure 3e,f). Orange needles (ON, Figure 5) were distinguished by their characteristic orange color and Raman signature (ν_CN_ = 2224 cm^–1^, Figure 4). Several yellow polymorphs Y04 (ν_CN_ = 2222 cm^–1^), YT04 (ν_CN_ = 2224 cm^–1^), and Y19 (ν_CN_ = 2224 cm^–1^) have similar Raman shifts but can be ruled out because they are neither orange nor needles commonly associated with these polymorphs. Orange plates (OP) were identified from their Raman signature, ν_CN_ = 2226 cm^–1^, and distinguished from R by their color despite similar morphologies (Figure 5). OP was seldom observed on 1% fibers and only immediately after extrusion. OP was observed after up to one month on 0.1% fibers, however. Plates of R (Figure 5) were identified based on their red color and ν_CN_ = 2211 cm^–1^ (Figure 4). Red plates, unlike those characteristic of R, exhibited a broader Raman peak than R, with ν_CN_ = 2214 cm^–1^ (Figure 4), and cannot be assigned unambiguously from values of ν_CN_ from the literature.^53^ The nearest match is the RPL form (the so-called red plate form), which has been reported to exhibit two peaks at ν_CN_ = 2210 and 2215 cm^–1^. Although this match is not perfect, the broader Raman feature may be masking the individual peaks.^72^

The SEM images and Raman spectra (Figure 4) of 1% ROY PE fibers reveal the formation of Y at the expense of the higher energy polymorphs ON, OP, and R. At the time of extrusion, the fibers containing 1% ROY were red, changing to orange over three months and eventually to yellow. Metastable forms were more persistent on 0.1% fibers than on 1% fibers as metastable phases R, ON, and OP persisted even after 28 days. Raman spectroscopy revealed that as the fibers aged, the crystals on the surface grew and/or transformed to the lower energy polymorphs, i.e., from R to polymorph Y, a demonstration of Ostwald’s rule of stages.^73^

**Figure 6:**
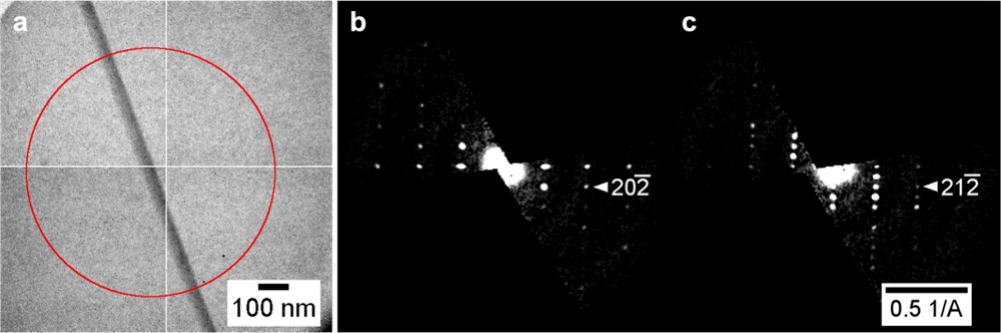
(a) 40 nm wide ON needle (QAXMEH54^59^) characterized by 3D ED with an electron beam of 800 nm diameter (red circle). Reciprocal space sections (b) *h*0*l* and (c) *h*1*l* of the 40 nm thin ON needle.

The polymorphic composition identified by Raman microscopy was also confirmed by 3D ED. The lattice constants of Y, ON, OP, and R polymorphs were determined and matched to known structures in the Cambridge Crystallographic Database.^51,52^ 3D ED is a rapid method to establish polymorphism statistics. For example, the polymorph composition (based on 33 crystals measured) on 0.1 wt % fibers after 6 days of aging was determined to be 2/33 Y, 2/33 R, and 29/33 ON. The characterization of a thin needle of ON is shown in Figure 6. The needle has a cross section of approximately 40 nm and was measured with an 800 nm diameter beam (Figure 6a). The irradiated volume of the crystal was approximately 0.001 μm^3^, and the data were collected over 108 s. Data completeness was 81%, and resolution was 1.0 Å^–1^. ON is the best match for the lattice parameters (*a* = 3.941(5), *b* = 18.47(4), c = 16.30(4) Å, β = 92.8(2)°).^53^ Moreover, these values, together with the systematic absences due to the *c*-glide plane (Figure 6b,c), led to the unambiguous assignment of the ON polymorph, (CSD Refcode QAXMEH54).^59^ We added this refinement to the CSD (deposition number 2330038) as it is the first ON determination by 3D ED.

**Figure 7:**
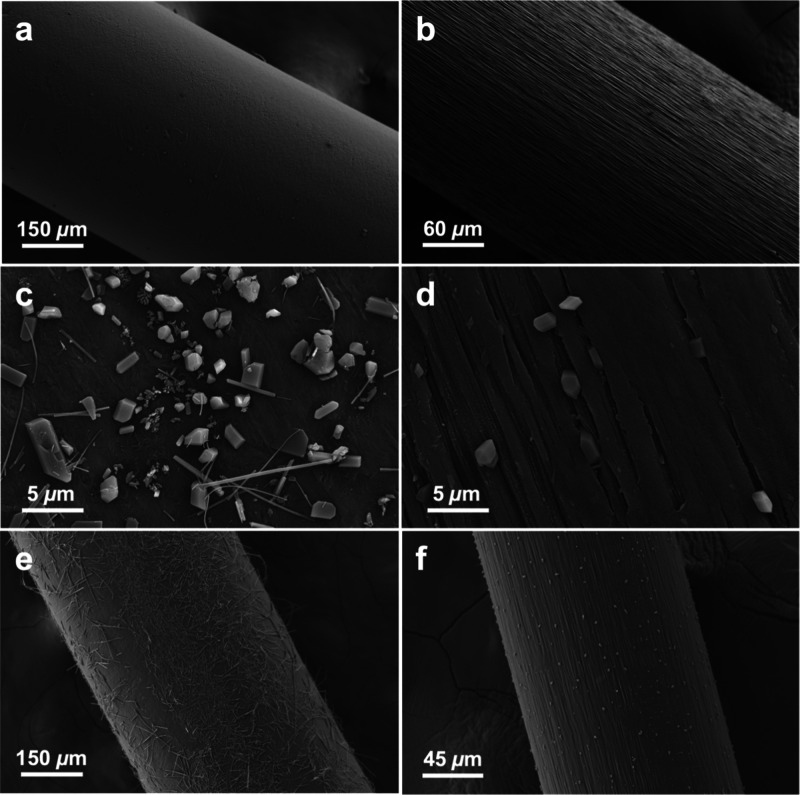
(a, c, e) SEM images of 1% fiber on days 1, 8, and 18, respectively. (b, d, f) SEM images of stretched 1% ROY fiber on days 1, 8, and 18, respectively.

PE is a semicrystalline material, consisting of both crystalline and amorphous regions. The ratio between these regions has an impact on blooming, although the associated molecular mechanisms are still being debated.^74−76^ Post-manufacturing processing can also have a significant influence on the diffusivity of blooming molecules and blooming rates through the reorganization of crystalline and amorphous domains.^1^ When stretched at room temperature, a procedure known as cold drawing, PE “necks” and spherulitic regions begin to reorient.^77^ Beyond the necked region, crystalline lamellae align with the drawing direction, which is evident from the change in the surface texture of the fiber (Figure 7a,b).

The cold drawing process has a significant effect on the polymorphs of ROY observed on 1% ROY fibers. The surface of as-extruded fibers is relatively smooth, and a diverse polymorphic population is observed (Figure 7c). Crystal morphology is unrestricted, and ROY crystallites sprawl across the surface (Figure 7e). On the other hand, stretched fibers have rougher surfaces because of grooves created by the alignment of PE lamellae. Crystals predominantly formed within these grooves, and as such their morphologies and polymorphic identity were templated. On stretched fibers, block and plate morphologies dominated and needles constituted a negligible proportion of the population. Crystallite sizes were also dramatically limited and more uniform. After 8 days, most crystals on stretched fibers were smaller than ca. 3 μm (Figure 7d), and almost all crystals were the Y or YN polymorphs. After 18 days, the crystals continued to grow in grooves, with some spilling out, and the surface remained dominated by Y and YN (Figure 7f). In contrast, after 8 days, as-extruded fibers featured a rich and diverse polymorphic population with many morphologies (Figures 3d and 7c). By day 18, the as-extruded fiber was covered in needles (some >100 μm in length) as well as other crystal morphologies (Figure 7e).

The rate of blooming was slower for stretched fibers; after approximately 150 days, the proportion of ROY that bloomed to the surface was ∼60% less than expected when compared to an as-extruded fiber and normalized for the change in fiber diameter. This may be explained by a reduction in polymer crystallinity and polymer-chain mobility that is imparted by the stretching and resulting alignment of lamellae. The color of the stretched fiber did not change per what was observed for as-extruded fibers, and in fact, stretched fibers remained red for the duration of the observation time.

**Figure 8:**
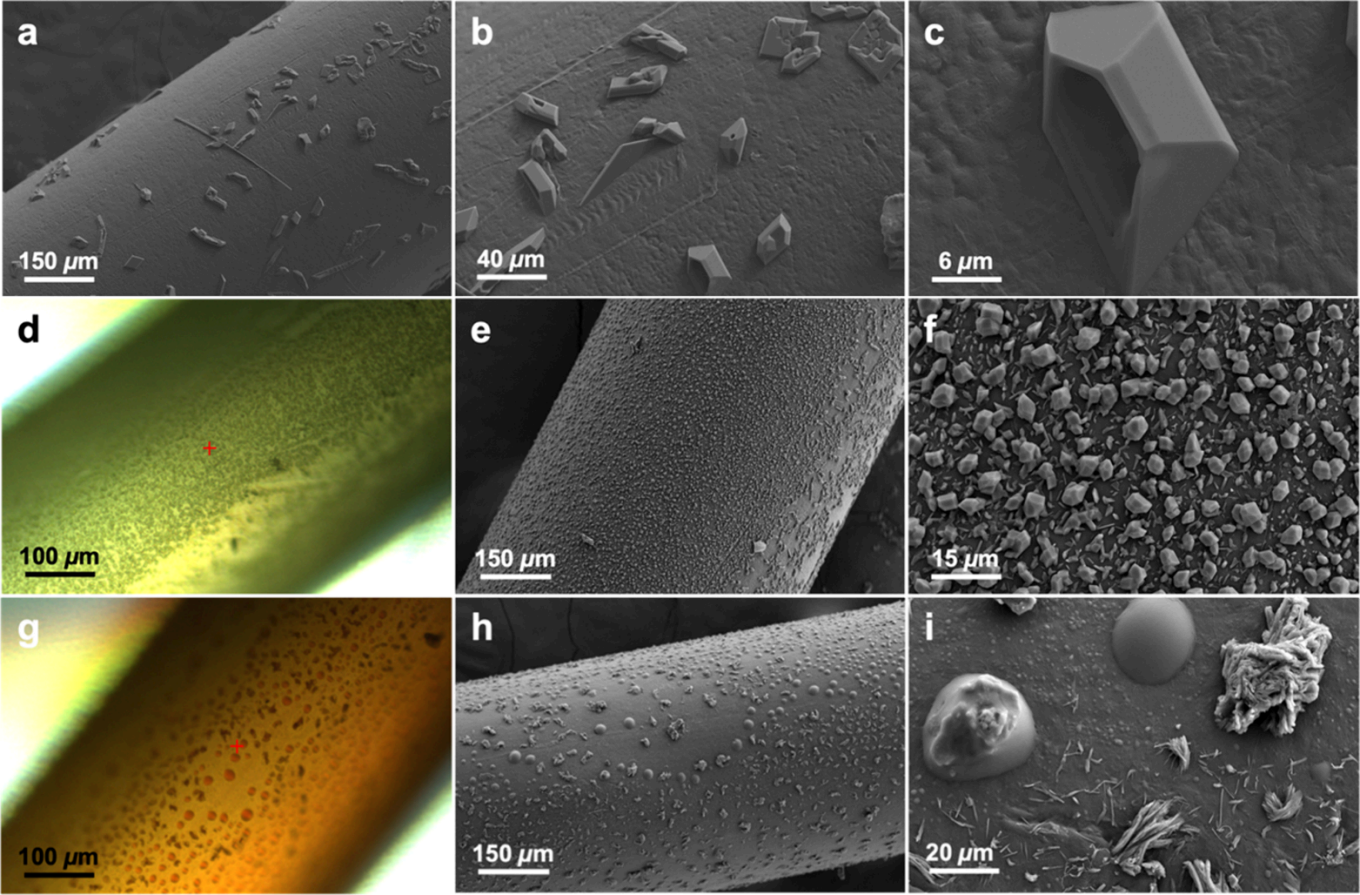
(a–c) SEM images of 1% ROY PE fiber washed weekly, 2 months after final wash, at progressively increasing magnification. (d) Optical micrograph of fiber aged for 5 months, (e, f) SEM images of fiber aged for 5 months, (g) optical micrograph of fiber after heating above ROY melting points (115 °C), and (h, i) SEM images of heated fiber.

The importance of diffusion for ROY crystallization on the fiber surface becomes very apparent when ROY concentration in the volume is small either because of a low initial concentration or because of fiber depletion after blooming. In this case, crystals develop hopper morphologies, with edges fully developed at the expense of intererior spaces, characteristic of diffusion-limited crystallization (Figure 8a–c). Heating aged fibers beyond the melting point of ROY restored the red color characteristic of newly extruded fibers (Figure 8g). Optical micrographs and SEM images reveal that the thermodynamically stable block-shaped Y after melting (*T*_m_ = 109.8 °C^78^) leaves droplets accompanied by newly crystallizing metastable forms emerging from equivalent droplets (Figure 8g–i). This observation demonstrates the feasibility of generating metastable forms of compounds on the surface of PE fibers, which is essential to improving the effectiveness of PE LLINs by generating more active metastable polymorphs on their surfaces.

## Conclusions

Long-lasting insecticidal bed nets made of poly(ethylene) fibers with contact insecticide embedded in the polymer at the time of manufacture function by insecticide molecules diffusing from within the supersaturated polymers to surfaces where they crystallize and become bioavailable to insects. Using the well-characterized ROY as a proxy for insecticide blooming and crystal growth on fibers, we have demonstrated that a compound that is highly polymorphic, like many contact insecticides, forms at least six of total 13 crystalline forms on the surface of PE fibers. The kinetic profile of blooming is consistent with crystallization at the fiber surface limited by the diffusion of ROY molecules from the fiber interior, and the observation of many polymorphs reveals that crystallization on fiber surfaces is complex and dynamic. We also have demonstrated how polymorphism can be controlled by post-extrusion processes and that metastable forms may be generated by thermal treatment. This study has articulated a framework for analyzing and characterizing insecticides that are released from PE bed nets. The methods described in the research can be used to identify different crystal forms, which can help improve vector control products through polymorph engineering. The framework can be applied in the field of vector control to gain a better understanding of the insecticide release process and ultimately develop more effective and efficient methods for combating diseases transmitted by insects.
